# Supplementary material for: Virally programmed extracellular vesicles sensitize cancer cells to oncolytic virus and small molecule therapy
Source: Nat Commun. 2022 Apr 7;13:1898. doi: 10.1038/s41467-022-29526-8 (PMC8990073; doi:10.1038/s41467-022-29526-8)
Supplement: Supplementary file 1 — Supplementary Information [file 41467_2022_29526_MOESM1_ESM.pdf]

SUPPLEMENTARY INFORMATION

Virally programmed extracellular vesicles sensitize cancer cells to oncolytic virus and small molecule therapy

Marie-Eve Wedge[1,2,a], Victoria A. Jennings[1,3,4,a], Mathieu Crupi[1,5a], Joanna Poutou[1,5a], Taylor Jamieson[1,5], Adrian Pelin[1,5], Giuseppe Pugliese[1], Christiano Tanese de Souza[1], Julia Petryk[1], Brian J. Laight[1], Meaghan Boileau[1], Zaid Taha[1,5], Nouf Alluqmani[1,5], Hayley E. McKay[1,5], Larissa Pikor[1], Sarwat Tahsin Khan[1], Taha Azad[1,5], Reza Rezaei[1,5], Bradley Austin[1], Xiaohong He[1], David Mansfield[3], , Elaine Rose[1,5], Emily E.F. Brown[1], Natalie Crawford[1], Almohanad Alkayyal[1,6], Abera Surendran[1,5], Ragunath Singaravelu[1,5], Dominic G. Roy[1,5], Gemma Migneco[4], Benjamin McSweeney[1], Mary Lynn Cottee[1], Egon J. Jacobus[1,7], Brian A. Keller[1,5], Takafumi N. Yamaguchi[8], Paul C. Boutros[8,9,10,11], Michele Geoffrion[12], Katey J. Rayner[5,12], Avijit Chatterjee[13], Rebecca C. Auer[1,5,14], Jean-Simon Diallo[1,5], Derrick Gibbings[2], Benjamin R. tenOever[15], Alan Melcher[3], John C. Bell[1,5] and Carolina S. Ilkow[1,5*]

[1]Centre for Innovative Cancer Therapeutics, Ottawa Hospital Research Institute, Ottawa, Ontario, Canada, [2]Department of Cellular and Molecular Medicine, University of Ottawa, Ottawa, Ontario, Canada, [3]Institute of Cancer Research, London, UK, [4] Leeds Institute of Medical Research at St James's, University of Leeds, Leeds, UK, [5]Department of Biochemistry, Microbiology and Immunology, University of Ottawa, Ottawa, Ontario, Canada [6]Department of Medical Laboratory Technology, Faculty of Applied Medical Sciences, University of Tabuk, Tabuk, Saudi Arabia, [7]Department of Oncology, University of Oxford, Oxford, UK, [8] Jonsson Comprehensive Cancer Center, University of California, Los Angeles, Los Angeles, [9]Department of Urology, University of California, Los Angeles, Los Angeles, CA, USA, [10]Institute for Precision Health, University of California, Los Angeles, Los Angeles, CA, USA, [11] Department of Human Genetics, University of California, Los Angeles, Los Angeles, CA, USA, [12]University of Ottawa Heart Institute, Ontario, Canada, [13]The Ottawa Hospital, Division of Gastroenterology, Ottawa, Ontario, Canada, [14]Department of Surgery, University of Ottawa, Ottawa, Canada, [15]Department of Microbiology, Icahn School of Medicine at Mount Sinai, New York, New York, USA

[a]These authors contributed equally to this work

[*]Corresponding author email: cilkow@uottawa.ca

This file contains eight supplementary figures and figure legends, and six supplementary tables.

Supplementary Figure 1

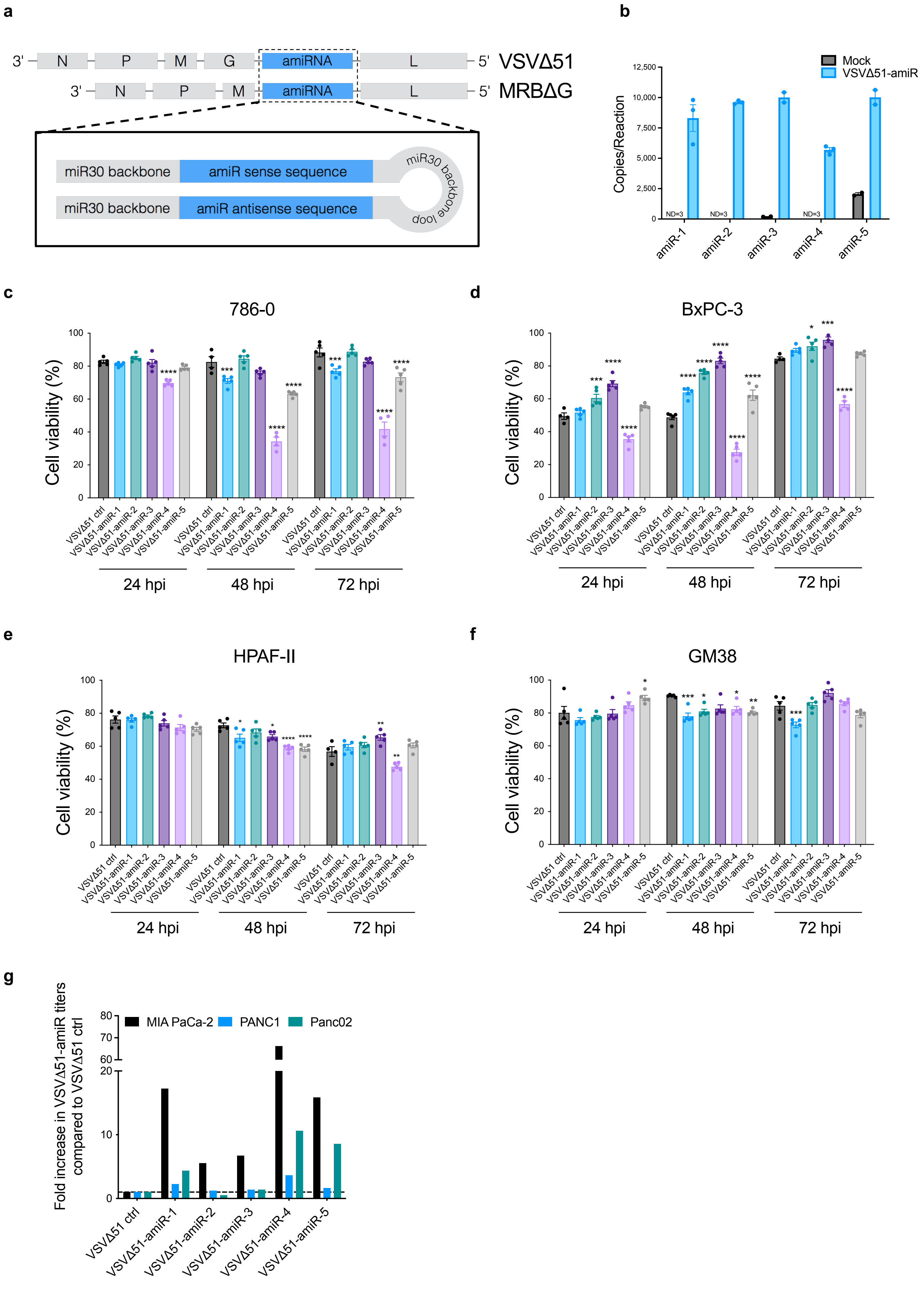

Supplementary Figure 1. **Functional expression of select artificial microRNAs from a VSVΔ51 oncolytic rhabdovirus platform enhances viral replication and cytotoxicity in cancer cells.**

(**a**) Schematic of the VSVΔ51 and MRBΔG genomes encoding amiRNA or shPD-L1 sequences built in the pre-miR-30 backbone. (**b**) Quantitative RT-PCR analysis of VSVΔ51-encoded amiR-1 to -5. Copies per reaction based on a relative standard curve were calculated and plotted. Error bars represent mean values ± SEM of 2 replicates (VSVΔ51 encoding amiR-3 and -5) or 3 replicates (VSVΔ51 encoding amiR-1, -2, and -4). ND=not detected. (**c-f**) Multiple cancer cell lines were infected with VSVΔ51-amiR-NTC (control) or VSVΔ51-amiR-1 to -5 at different MOIs. Specifically, (**c**) 786-0 cells were infected at MOI 1, (**d**) BxPC-3 at MOI 5, (**e**) HPAF-II at MOI 3, and (**f**) GM38 at MOI 1. Cell viability was measured by alamarBlue® Assay at indicated time points and compared to virus control. Error bars represent mean values ± SEM for 4 biological replicates (786-0: VSVΔ51 ctrl at 48 h, VSVΔ51-amiR-4 at 48 h and 72 h; BxPC-3: VSVΔ51 ctrl at 24 h and 48 h, VSVΔ51-amiR-4 at 72 h; HPAF-II: VSVΔ51 ctrl at 72 h), or 5 biological replicates per condition (other groups). Two-way ANOVA with Tukey's multiple comparison test (95% confidence intervals [CI]); adjusted p-values: * p<0.05, ** p<0.01, *** p<0.001, **** p<0.0001. Exact p-values are provided in the Source data file. (**g**) Fold increase in VSVΔ51-amiR-4 titers relative to VSVΔ51-amiR-NTC in MIA PaCa-2, PANC1, and Panc02 cells (48 hpi [hours-post infection] at MOI 0.1). n=1 per condition. Source data are provided as a Source data file.

Supplementary Figure 2

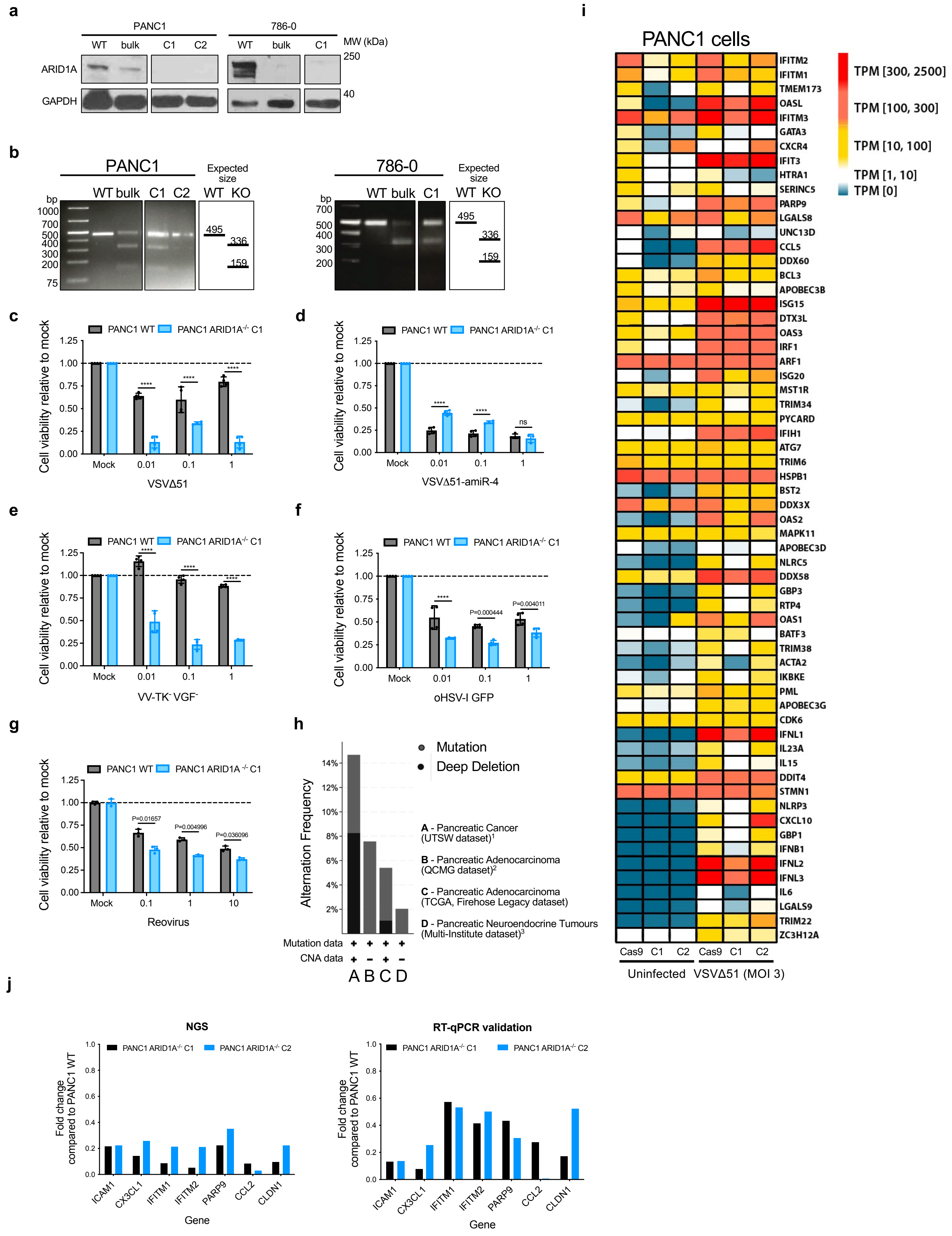

Supplementary Figure 2. ***ARID1A*** **depletion sensitizes cancer cells to multiple oncolytic viruses.**

(**a**) Immunoblotting analysis of *ARID1A* CRISPR-Cas9 knockouts of PANC1 and 786-0 bulk populations and clonal cell populations. GAPDH is included as a loading control (n=2). Uncropped images are shown in Supplementary Fig. 8. (**b**) T7 assay of wild-type and *ARID1A* knockout PANC1 and 786-0 cells on genomic DNA. To check for *ARID1A* targeting using the CRISPR-Cas9 system, the expected target region was amplified using specific primer sets and a T7 assay was performed with 3 technical replicates. PCR products were resolved by agarose gel electrophoresis. The size of the expected amplicons is indicated. (**c-g**) PANC1 WT and *ARID1A*$^{-/-}$ cells were infected with VSVΔ51-GFP (**c**), VSVΔ51-amiR-4 (**d**), VV-TK$^-$VGF$^-$ (**e**), oHSV-1-GFP (**f**), or Reovirus (**g**) at the indicated MOIs. AlamarBlue® cytotoxicity assay quantification 48 hpi (VSVΔ51-GFP and VSVΔ51-amiR-4) or 24 hpi (VV-TK$^-$VGF$^-$ and oHSV-1-GFP) compared to their corresponding uninfected conditions are shown as mean values ± SEM (n=4 for VSVΔ51-GFP, VSVΔ51-amiR-4, VV-TK$^-$VGF$^-$, oHSV-1-GFP and n=3 for Reovirus). Two-way ANOVA with Sidak's multiple comparisons test (95% CI); adjusted p-values: ns $p>0.05$, **** $p<0.0001$. (**h**) Frequency of *ARID1A* mutation or deep deletion in pancreatic cancers. Data were obtained and plotted using the cBioPortal for Cancer Genomics (http://cbioportal.org). (**i**) Heatmap showing transcript expression levels (Log2 TPM [transcripts per kilobase million]) of anti-viral genes in uninfected PANC1 wild-type or *ARID1A*-deficient cells. Note that two independent clones for *ARID1A*-deficient cells were included (C1 and C2). (**j**) RT-qPCR validation analysis of differentially expressed genes involved in immune function that were found to be downregulated in *ARID1A*$^{-/-}$ PANC1 cells compared to wild-type by next-generation sequencing (NGS). Analysis was conducted in two different clonal populations of *ARID1A*$^{-/-}$ PANC1 clone 1 (C1) and clone 2 (C2) cells. Fold change in gene expression was calculated relative to its expression level in wild-type cells. Bars represent average values for 2 biological replicates per condition. Source data are provided as a Source data file.

Supplementary Figure 3

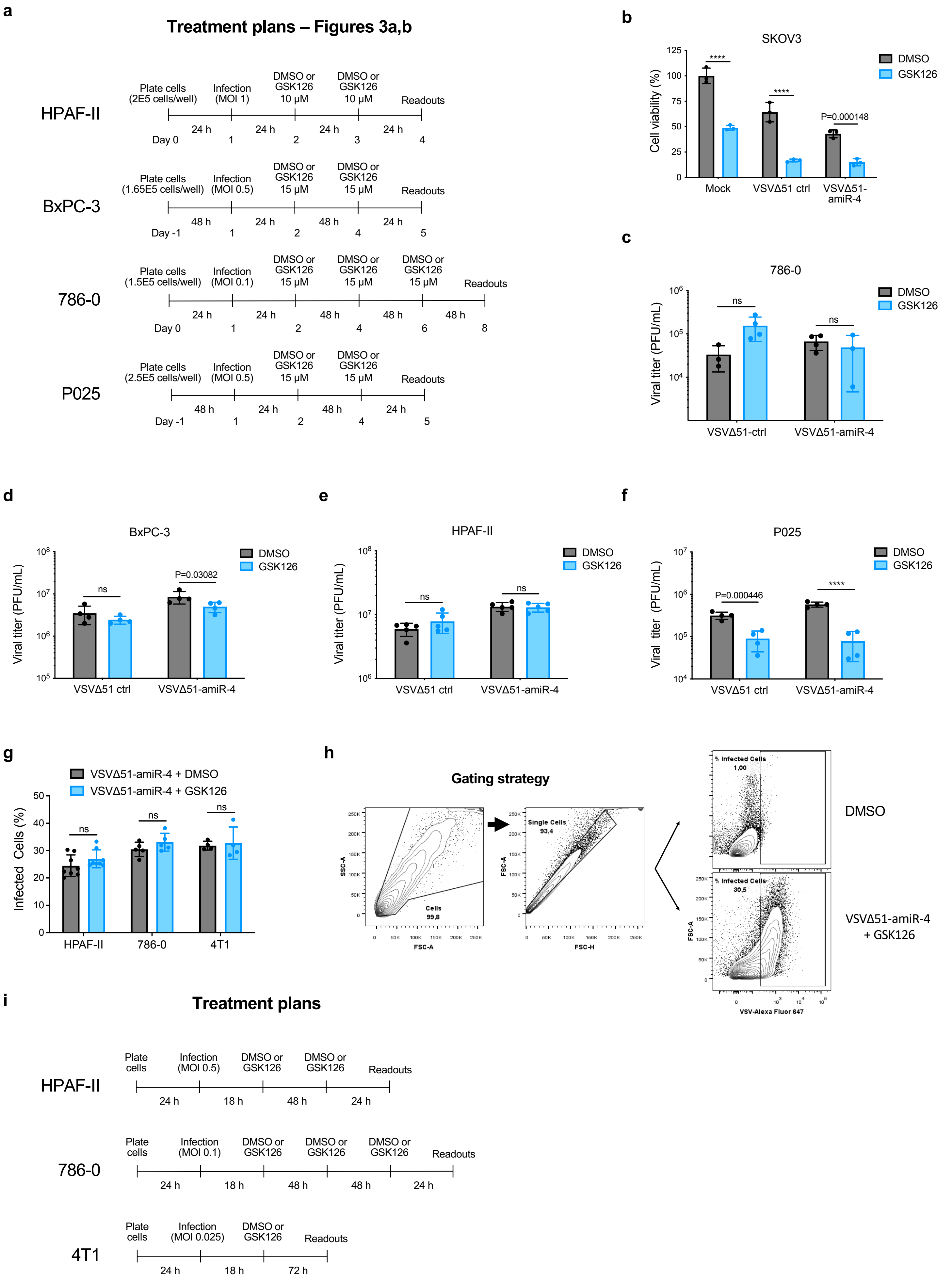

Supplementary Figure 3. The combination of VSVΔ51-amiR-4 and GSK126 enhances cancer cell death.

(**a**) Experimental outlines of VSVΔ51-amiR-4 and GSK126 treated cell lines in Fig. 3a,b and Supplementary Figure 3 c-f are shown. (**b**) Cell viability in *ARID1A*-deficient and GSK126-sensitive SKOV-3 cells following VSVΔ51-amiR-4 infection and GSK126 treatment shows no advantage of the combinatorial approach compared to uninfected DMSO treated cells. Error bars represent mean values ± SEM for 3 biological replicates per condition. Two-way ANOVA with Sidak's multiple comparisons test (95% CI); adjusted p-values: **** p<0.0001. (**c-f**) Viral titers of infected cells at readout following a synthetic lethality treatment plan displayed in **a**. Error bars represent mean values ± SEM for 5 biological replicates per condition (HPAF-II), 4 biological replicates per condition (BxPC-3, P025, 786-0 with VSVΔ51 ctrl and GSK126, or with VSVΔ51-amir-4 and DMSO) or 3 biological replicates per condition (786-0 with VSVΔ51-amir-4 and GSK126, or with VSVΔ51 ctrl and DMSO). Two-way ANOVA with Sidak's multiple comparisons test (95% CI); adjusted p-values: ^{ns} p>0.05, **** p<0.0001. (**g**) Flow cytometry assessment of infected cancer cells with or without GSK126 treatment (as outlined in **i**). Two-way ANOVA with Sidak's multiple comparisons test (95% CI); adjusted p-values: ^{ns} p>0.05. Error bars represent mean values ± SEM of biological replicates (HPAF: n=8 per condition; 786-0: n=5 per condition; 4T1: n=4 per condition). (**j**) Gating strategy used for sample analysis in **g** with 4T1 data is used as an example. (**i**) Experimental outlines of VSVΔ51-amiR-4 and GSK126 treated cell lines in Supplementary Fig. 3j are shown. Source data are provided as a Source data file.

Supplementary Figure 4

a

786-0 SEV concentration	
Sample	Concentration (particles/mL)
Mock	1.16E09
MRBΔG-amiR-NTC	4.03E10
MRBΔG-amiR-4	3.81E10

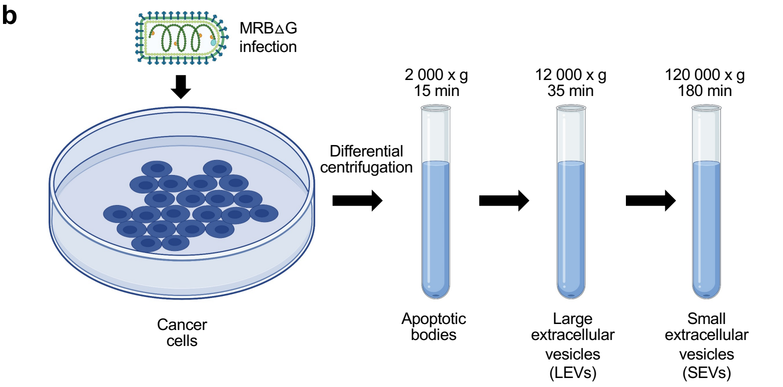

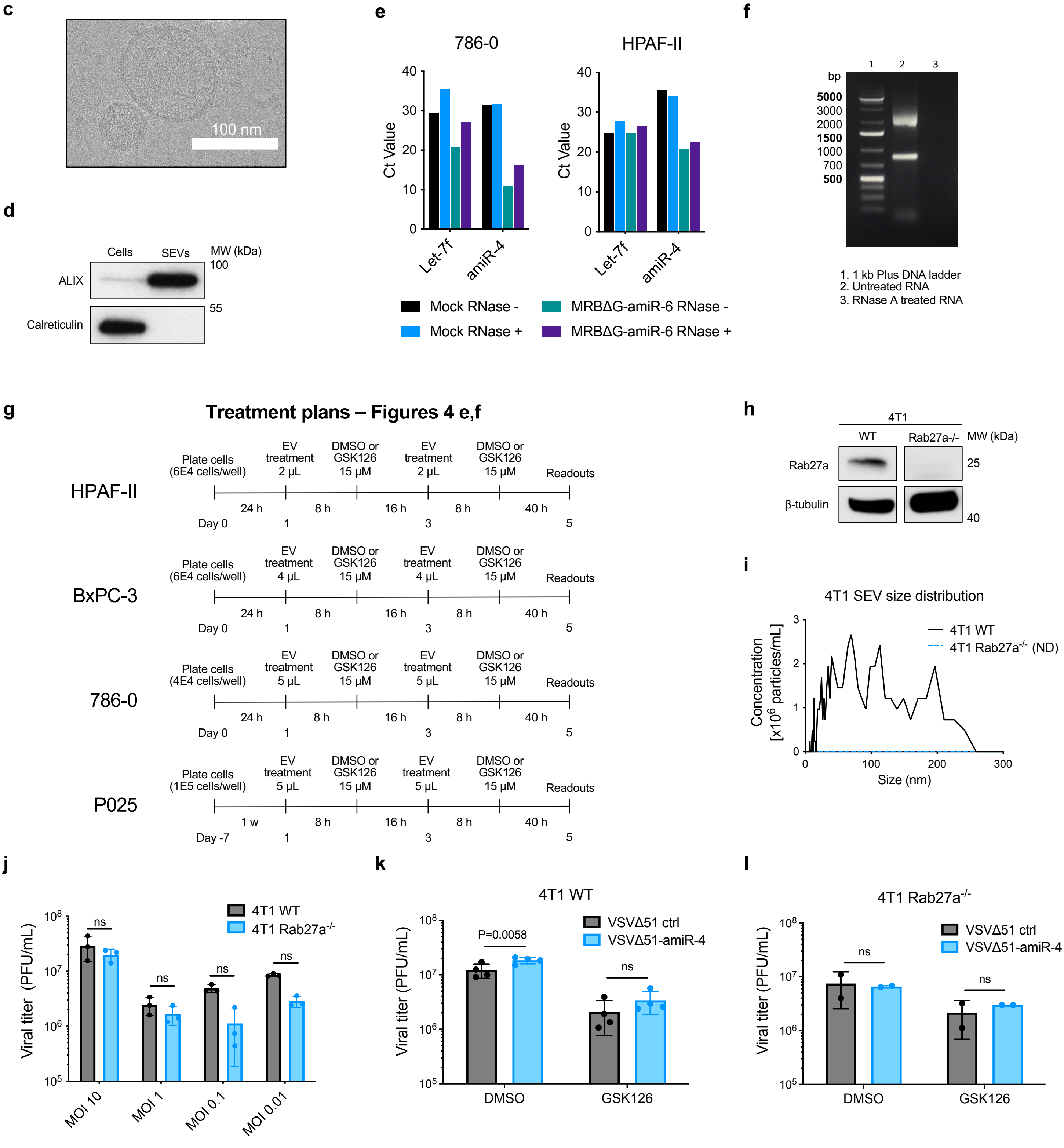

Supplementary Figure 4. **Characterization of SEVs produced by oncolytic rhabdovirus-infected cells.**

(**a**) Representative nanoparticle tracking analysis (NTA) quantification of SEVs produced from mock-infected or MRBΔG-infected 786-0 cells (MOI=5, n=2). (**b**) Schematic representation of the SEV harvesting procedure from G-deficient MRB-infected cells (created using BioRender). (**c**) Representative electron microscopy images of SEVs harvested from MRBΔG-infected Mel888 cells at MOI 1 (n=2). Scale bar = 100 nm. (**d**) Immunoblotting analysis for ALIX (SEV marker) and calreticulin (specific cell-associated marker) in purified SEVs and whole Mel888 cell lysates (n=3). Uncropped images are shown in Supplementary Fig. 8. (**e**) 786-0 and HPAF-II cells were left uninfected or were infected with MRBΔG-amiR-4 (MOI=1). SEVs were collected by ultracentrifugation 48 hpi, and an RNase protection assay was performed to remove free, non-encapsulated RNA. SEV-associated amiR-4 levels were then quantified by RT-qPCR analysis (n=2). (**f**) RNase A treated 786-0 cellular RNA were also resolved by agarose gel electrophoresis (n=2). (**g**) Experimental outlines of SEV-educated and GSK126-treated cell lines in Fig. 4e,f are shown. (**h**) Immunoblotting analysis of 4T1 wild-type and *Rab27a* CRISPR-Cas9 knockout clonal cell line. β-tubulin is included as loading control (n= 2). (**i**) NTA quantification and size distribution profiles of SEVs produced by 4T1 WT and Rab27a$^{-/-}$ cells. ND=not detected (below detection limit). Representative of biological triplicates. (**j**) 4T1 WT or Rab27a$^{-/-}$ cells produce similar amounts of virus following VSVΔ51 infection at indicated MOIs as quantified by plaque assay. Error bars represent mean values ± SEM of 3 biological replicates. Two-way ANOVA with Sidak's multiple comparison test (95% CI), ^{ns} p>0.05. (**k,l**) 4T1 WT or Rab27a$^{-/-}$ cells infected with VSVΔ51 or VSVΔ51-amiR-4 (MOI 0.025) and treated with vehicle control (DMSO) and GSK126 (15 μm) for 72 h. The production of infectious particles for each condition was quantified by plaque assay. Data represent mean values ± SEM of 4 biological replicates for wild-type cells and two for Rab27a$^{-/-}$ cells. For 4T1 WT, two-way ANOVA with Sidak's multiple comparison test (95% CI), adjusted p-values: ^{ns} p>0.05. Source data are provided as a Source data file.

Supplementary Figure 5

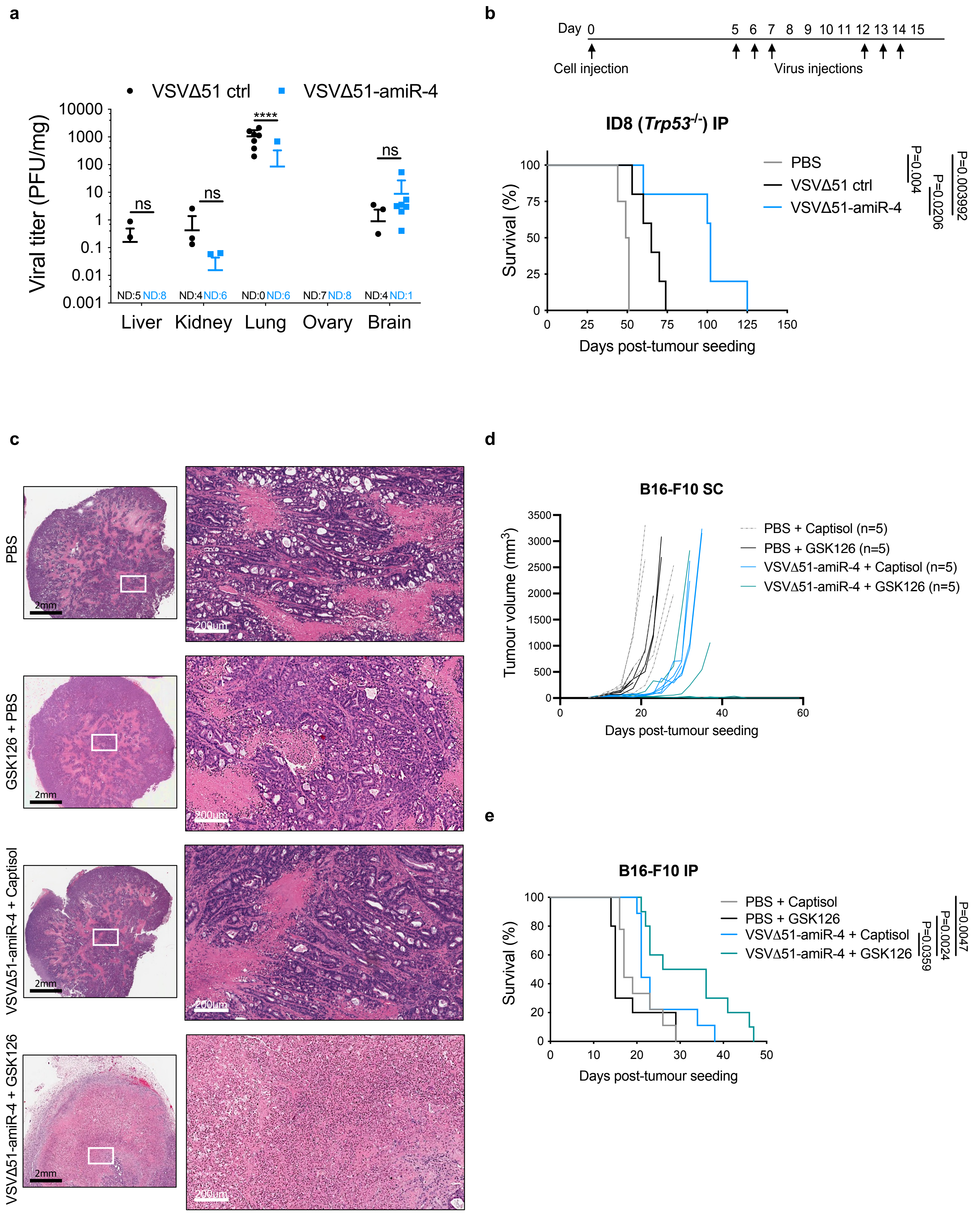

Supplementary Figure 5. amiR-4 does not alter the biodistribution of VSVΔ51 but enhances survival of tumour-bearing immunocompetent murine intraperitoneal models.

(**a**) Quantification of VSVΔ51-amiR-NTC or VSVΔ51-amiR-4 infectious particles from different organs obtained from mice intravenously (IV)-treated with the indicated viruses (1E8 pfu/mice) for 48 h. Data represent mean values ± SEM of 7 independent biological replicates for VSVΔ51-amiR-NTC and 8 independent biological replicates for VSVΔ51-amiR-4. Two-way ANOVA with Sidak's multiple comparisons test (95% CI); ^{ns} p>0.05, **** p<0.0001. ND=not detected. (**b**) Kaplan-Meier survival analysis of orthotopic ID8 *Trp53*$^{-/-}$ peritoneal carcinomatosis-bearing mice treated with six IP doses of PBS (n=4), VSVΔ51-amiR-NTC (n=5) or VSVΔ51-amiR-4 (n=5). Log-rank (Mantel-Cox) test. (**c**) Representative images of HPAF II tumours stained with Hematoxylin and Eosin (n=4 per condition). Tumour-bearing mice were treated as shown in Figure 5f and tumours were collected and stained at the experimental endpoint. The enlarged image sections (right images, scale bar=200 μm) are indicated by white rectangles (left images, scale bar=2 mm). (**d**) Mice bearing subcutaneous B16-F10 tumours were treated as indicated with vehicle controls (PBS and/or Captisol) or with VSVΔ51-amiR-4 or GSK126 (50 mg/kg) or the combination of both monotherapies. Individual tumour growth curves of mice are shown (n=5 per group). (**e**) Kaplan-Meier survival curves of mice bearing intraperitoneal B16-F10 tumours and treated as indicated with vehicle controls (PBS and/or Captisol) or with VSVΔ51 control or VSVΔ51-amiR-4 or GSK126 (50 mg/kg) or the combination of both monotherapies. Log-rank (Mantel-Cox) test, (n=10 for PBS + GSK126 and n=9 for both VSVΔ51-amiR-4 + GSK126 groups and VSVΔ51-amiR-4 + Captisol groups; data represents two pooled individual experiments). Source data are provided as a Source data file.

Supplementary Figure 6

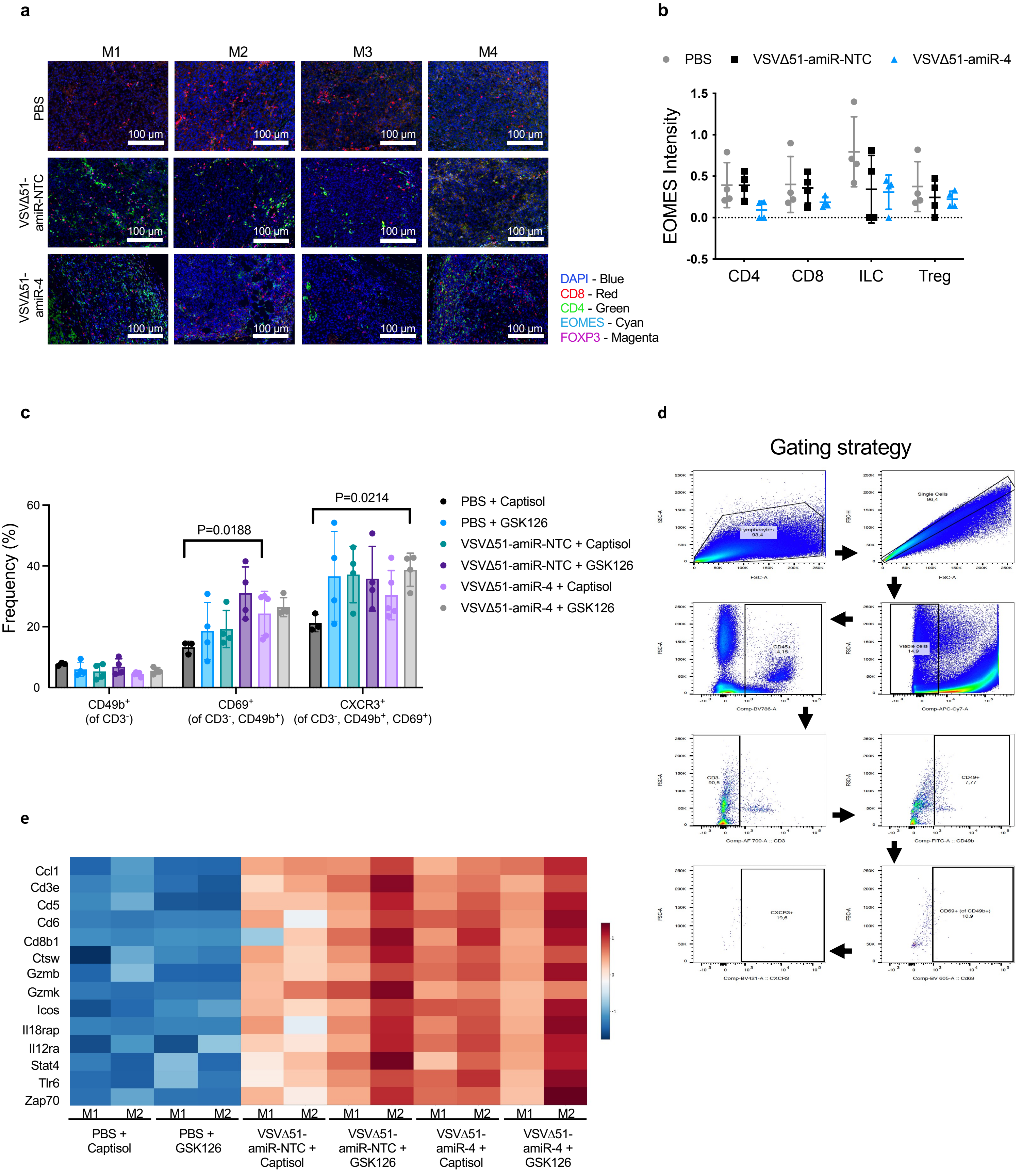

Supplementary Figure 6. Expression of amiR-4 from a VSVΔ51 platform did not induce drastic changes in the immune tumour microenvironment when compared to virus control in the presence or absence of GSK126 treatment.

(a,b) B16-F10 subcutaneous tumours were collected for immunophenotyping one- or five-days post-treatment of oncolytic virus and drug, as indicated in the Method section. (**a**) Multiplex immunohistochemistry of tumour samples treated with PBS, VSVΔ51-amiR4 or VSVΔ51-amiR-NTC control. Samples were stained for DAPI (blue), CD8 (red), CD4 (green), EOMES (cyan) and FOXP3 (magenta) (n= 4). Scale bar=100 μm (**b**) Multiplex immunohistochemistry quantification of EOMES intensities for CD4, CD8, ILC, Treg populations. Error bars represent mean values ± SEM for 4 biological replicates per condition. (**c**) Immunophenotyping of B16-F10 treated with PBS (vehicle control), VSVΔ51-amiR-NTC control and VSVΔ51-amiR-4 treatment (1E8 pfu/mouse) alone or in combination with Captisol or GSK126 (50 mg/kg). Isolated tumours were stained with extracellular markers as indicated in the Method section and Table 6 to profile immune cell populations (acute NK cell responses) and their activation status. Two-way ANOVA with Bonferroni's correction. Error bars represent mean values ± SEM for 3 biological replicates (PBS + Captisol), 5 biological replicates (VSVΔ51-amiR-4 + Captisol), or 4 biological replicates per condition (all other groups). (**d**) Gating strategy for flow cytometry experiment performed in c). (**e**) NanoString analyses of B16-F10 tumours (n=2 per condition) treated with PBS (vehicle control), VSVΔ51-amiR-NTC control and VSVΔ51-amiR-4 treatment (1E8 pfu/mouse) alone or in combination with Captisol or GSK126 (50 mg/kg). Full NanoString data set is included in Supplementary Data 2. The colour scale corresponds to relative gene expression levels based on Z-score analysis. Source data are provided as a Source data file.

Supplementary Figure 7

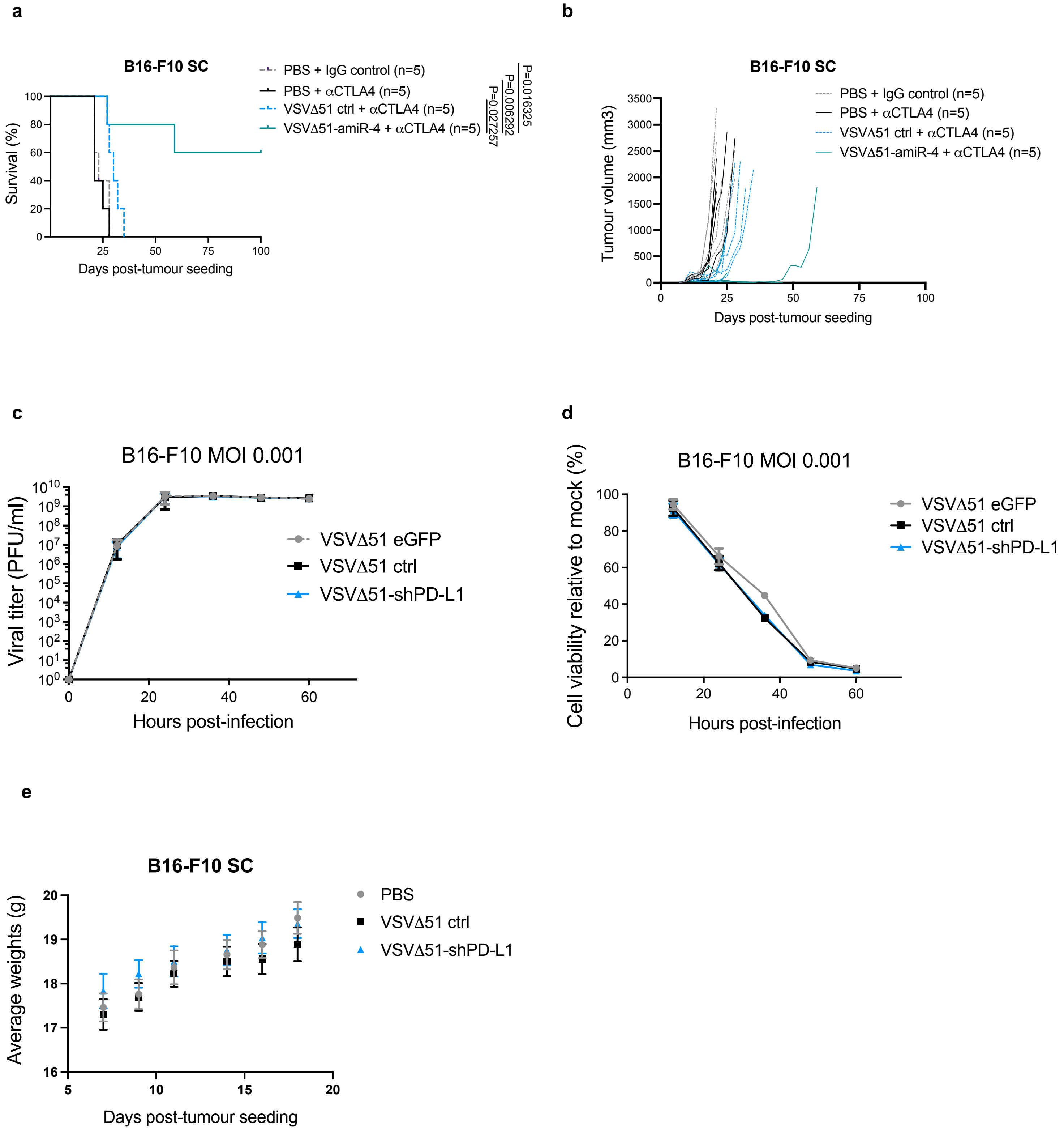

Supplementary Figure 7. Targeting immune checkpoints with novel oncolytic viruses.

(**a**,**b**). Kaplan-Meier survival curves (**a**) and Individual tumour growth curves (**b**) of mice bearing subcutaneous (SC) B16-F10 tumours and treated as indicated with vehicle controls (PBS and/or IgG) or with VSVΔ51 control or VSVΔ51-amiR-4 or anti-CTLA4 (50 mg/kg) or the combination of both monotherapies. Log-rank (Mantel-Cox) test (n=5 per group). **(c,d)** Multi-step growth curves (**c**) and cytotoxicity assays (**d**) were conducted in B16-F10 cells to evaluate growth and killing activity of VSVΔ51-shPD-L1 and VSVΔ51-amiR-NTC control (MOI=0.001). Data represent mean values ± SEM of 3 biological replicates. (**e**) Growth curves showing the mean body weight ± SEM of mice bearing subcutaneous B16-F10 tumours and treated as indicated with vehicle control (PBS) or with VSVΔ51-amiR-NTC control or VSVΔ51-shPD-L1. (n=10 mice per group). Two-way ANOVA with Sidak's multiple comparison test (95% CI), no statistical differences were found ($p>0.05$). Source data are provided as a Source data file.

Supplementary Figure 8

Supplementary Figure 2a

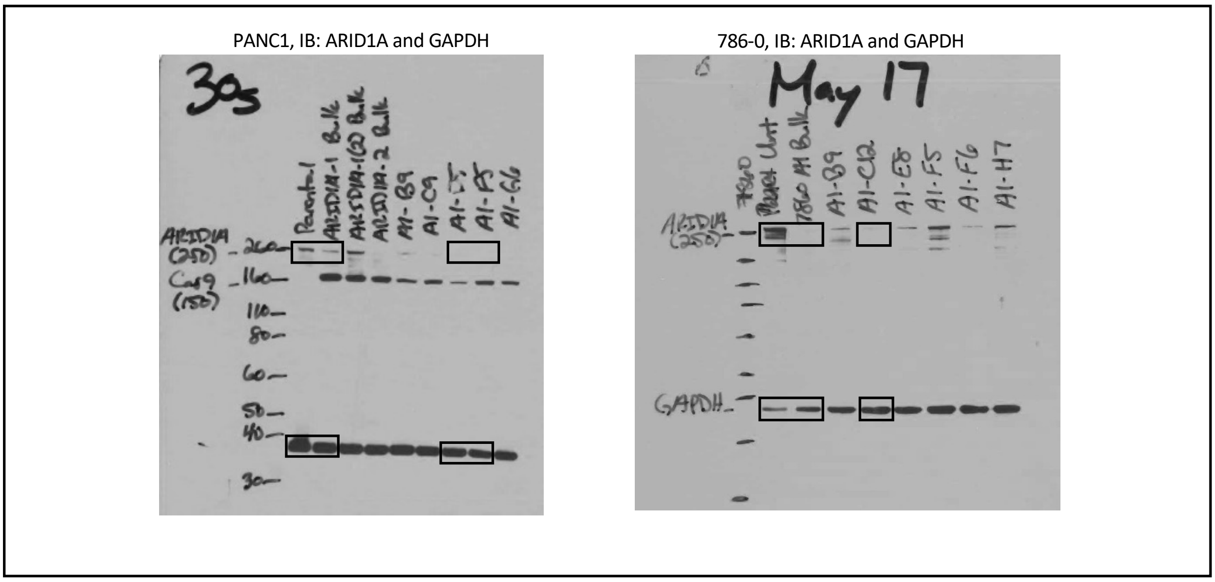

Supplementary Figure 4d

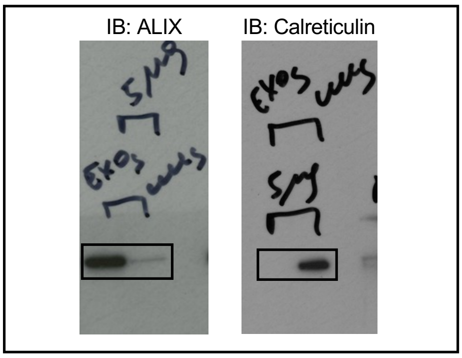

Supplementary Figure 4h

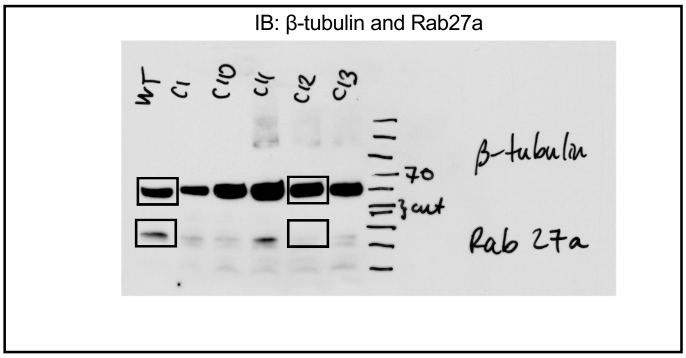

Supplementary Figure 8. Uncropped scans of western blots displayed in Supplementary Fig. 2a and Supplementary Fig. 4d,h.

SUPPLEMENTARY TABLES

Supplementary Table 1. Artificial miRNA sequences.

Artificial microRNA	Sense sequence (5'-3')	Antisense sequence (5'-3')
amiR-1	TTGTCTTACTCTTCAATAACAT	ATGTTATTGAAGAGTAAGACAA
amiR-2	ATAGTGATAACTCACTAGTACC	GGTACTAGTGAGTTATCACTAT
amiR-3	TTTAGTGATAACTCATAGTACA	TGTACTATGAGTTATCACTAAA
amiR-4	ACCGTCATGTCTGTTACGTTAA	TTAACGTAACAGACATGACGGT
amiR-5	TGCAGAGAGTGTTATATTGCAT	ATGCAATATAACACTCTCTGCA
amiR-NTC (GFP)	ACAAGCTGACCCTGAAGTTCAT	ATGAACTTCAGGGTCAGCTTGC
amiR-NTC (Fluc)	GTTGGCCACCGAAGCAGCGCAC	GTGCGCTGCTTCGGTGGCCAAC
amiR-shPD-L1	TTCAACACTGCTTACGTCTCCT	AGGAGACGTAAGCAGTGTTGAA

Supplementary Table 2. List of potential amiR-4 targets based on total energy of duplex as predicted by TargetS.

Gene Symbol	Gene Name	Duplex Free Energy
ARID1A	AT rich interaction domain 1A (SWI-like)	-77.1698
PLEC	Plectin	-74.52005
MCM2	Minichromosome maintenance complex component 2	-70.41588

Supplementary Table 3. List of potential amiR-4 targets based on BLAST complementarity.

Gene symbol	Gene name	Number of Complementary Bases	Range	Expect Value	Pos/Neg Strand
HDAC4	Histone deacetylase 4	16	2-17	5.0	Minus

Supplementary Table 4. Primers (Integrated DNA Technology) used for CRISPR knockout clones T7 assay and Sanger sequencing.

Targeted gene	Forward primer (5'-3')	Reverse primer (5'-3')
ARID1A - T7 assay	TGTGTGTGATACTGGGAGGT	CACAATTTGCTGCTGGGTCT
Rab27a - T7 assay	CTTAGCTCCTCCTTTTGTGC	TTCCTGAGAGGATGAGGAAG
ARID1A - sequencing	GCCTCTTCATGAGCCATTTC	AATTTGCTGCAGGGATTGTC
Rab27a - sequencing	CTCTTCCACCATACTTGGAG	TAGTAGCCTCGACACTGAGC

Supplementary Table 5. Primers (Integrated DNA Technology) used in RT-qPCR assays.

Gene or miRNA	Forward primer (5'-3')	Reverse primer (5'-3')
ARID1A	GAAGTGACTCCACATTCCAG	ACTCCCTGGAGCTTTCC
PLEC	CTGCACTTCCAGATCTCAG	CTGGAGGTGAAGTTGTCG
HDAC4	GACCTGACCGCCATTTGC	GGGAGAGGATCAAGCTCGTTT
MCM2	ATCAGAACTACCAGCGTATC	TCAGCTCTATCTCGTCTCC
ICAM-I	GGCTGACGTGTGCAGTAATA	CCTCTGGCTTCGTCAGAATC
CXC3L1	TGTAGCTTTGCTCATCCACTATC	CCTTGACCCATTGCTCCTT
IFITM1	ATCAACATCCACAGCGAGAC	GGAGTAGGCGAATGCTATGAAG
IFITM2	CCTGTTCAACACCCTCTTCAT	AACCATCTTCCTGTCCCTAGA
PARP9	GTACCTTGGGAGAAAGGAACAT	CGGGCTCCTTCAATCTCTAAC
CCL2	CTCAGCCAGATGCAATCAATG	TGCTGCTGGTGATTCTTCTAT
CLDN1	AAGTGCTTGGAAGACGATGAG	TACCATGCTGTGGCAACTAAA
Rplp0	TTAAACCCTGCGTGGCAATCC	CCACATTCCCCCGGATATGA
hsa-let-7f-1 5p	TGAGGTAGTAGATTGTATAGTT	PerfeCTa® Universal PCR Primer (QuantaBio qScript® MicroRNA cDNA Synthesis kit)

Supplementary Table 6. List of antibodies used for immune profiling by flow cytometry.

Marker	Fluorophore	Clone	Company, Catalogue	Dilution
CD45	BV786	30 F11	BD, 564225	1:1000
CD3	AF700	500A2	BD, 557984	1:50
CD69	BV605	H1.2F3	BD, 563290	1:100
NK1.1	APC	PK136	ThermoFisher Scientific, 17-5941-82	1:100
CD49b	FITC	DX5	BD, 561067	1:100
CD122	PE	5H4	BioLegend, 105906	1:100
CXCR3	BV421	CXCR3-173	BD, 562937	1:100

REFERENCES

1. Witkiewicz, A., McMillan, E., Balaji, U. *et al.* Whole-exome sequencing of pancreatic cancer defines genetic diversity and therapeutic targets. *Nat Commun* **6,** 6744 (2015).

2. Bailey P, Chang DK, Nones K, Johns AL, Patch AM, Gingras MC, Miller DK, Christ AN, Bruxner TJ, Quinn MC, Nourse C, Murtaugh LC, Harliwong I, Idrisoglu S, Manning S, Nourbakhsh E, Wani S, Fink L, Holmes O, Chin V, Anderson MJ, Kazakoff S, Leonard C, Newell F, Waddell N, Wood S, Xu Q, Wilson PJ, Cloonan N, Kassahn KS, Taylor D, Quek K, Robertson A, Pantano L, Mincarelli L, Sanchez LN, Evers L, Wu J, Pinese M, Cowley MJ, Jones MD, Colvin EK, Nagrial AM, Humphrey ES, Chantrill LA, Mawson A, Humphris J, Chou A, Pajic M, Scarlett CJ, Pinho AV, Giry-Laterriere M, Rooman I, Samra JS, Kench JG, Lovell JA, Merrett ND, Toon CW, Epari K, Nguyen NQ, Barbour A, Zeps N, Moran-Jones K, Jamieson NB, Graham JS, Duthie F, Oien K, Hair J, Grützmann R, Maitra A, Iacobuzio-Donahue CA, Wolfgang CL, Morgan RA, Lawlor RT, Corbo V, Bassi C, Rusev B, Capelli P, Salvia R, Tortora G, Mukhopadhyay D, Petersen GM; Australian Pancreatic Cancer Genome Initiative, Munzy DM, Fisher WE, Karim SA, Eshleman JR, Hruban RH, Pilarsky C, Morton JP, Sansom OJ, Scarpa A, Musgrove EA, Bailey UM, Hofmann O, Sutherland RL, Wheeler DA, Gill AJ, Gibbs RA, Pearson JV, Waddell N, Biankin AV, Grimmond SM. Genomic analyses identify molecular subtypes of pancreatic cancer. Nature. 2016 Mar 3;531(7592):47-52.

3. Scarpa A, Chang DK, Nones K, Corbo V, Patch AM, Bailey P, Lawlor RT, Johns AL, Miller DK, Mafficini A, Rusev B, Scardoni M, Antonello D, Barbi S, Sikora KO, Cingarlini S, Vicentini C, McKay S, Quinn MC, Bruxner TJ, Christ AN, Harliwong I, Idrisoglu S, McLean S, Nourse C, Nourbakhsh E, Wilson PJ, Anderson MJ, Fink JL, Newell F, Waddell N, Holmes O, Kazakoff SH, Leonard C, Wood S, Xu Q, Nagaraj SH, Amato E, Dalai I, Bersani S, Cataldo I, Dei Tos AP, Capelli P, Davì MV, Landoni L, Malpaga A, Miotto M, Whitehall VL, Leggett BA, Harris JL, Harris J, Jones MD, Humphris J, Chantrill LA, Chin V, Nagrial AM, Pajic M, Scarlett CJ, Pinho A, Rooman I, Toon C, Wu J, Pinese M, Cowley M, Barbour A, Mawson A, Humphrey ES, Colvin EK, Chou A, Lovell JA, Jamieson NB, Duthie F, Gingras MC, Fisher WE, Dagg RA, Lau LM, Lee M, Pickett HA, Reddel RR, Samra JS, Kench JG, Merrett ND, Epari K, Nguyen NQ, Zeps N, Falconi M, Simbolo M, Butturini G, Van Buren G, Partelli S, Fassan M; Australian Pancreatic Cancer Genome Initiative, Khanna KK, Gill AJ, Wheeler DA, Gibbs RA, Musgrove EA, Bassi C, Tortora G, Pederzoli P, Pearson JV, Waddell N, Biankin AV, Grimmond SM. Whole-genome landscape of pancreatic neuroendocrine tumours. Nature. 2017 Mar 2;543(7643):65-71. Epub 2017 Feb 15. Erratum in: Nature. 2017 Sep 27; PMID: 28199314.
