# Supplementary material for: Virally programmed extracellular vesicles sensitize cancer cells to oncolytic virus and small molecule therapy
Source: Nat Commun. 2022 Apr 7;13:1898. doi: 10.1038/s41467-022-29526-8 (PMC8990073; doi:10.1038/s41467-022-29526-8)
Supplement: Supplementary file 4 — Description of Additional Supplementary Files [file 41467_2022_29526_MOESM4_ESM.pdf]

Supplementary Data 1.

Description: A Sindbis virus-based amiRNA library was serially passaged in MIA PaCa-2 cells (n=5), PanC02 cells (n=5), PCa fibroblasts (n=5), and GM38 fibroblasts (n=5). Target RNA sequencing was conducted to elucidate top amiRNA sequences enriched upon selection. Full targeted RNA sequencing data of unpassaged Sindbis virus-based amiRNA library or library passaged is included.

Supplementary Data 2.

Description: Expression of amiR-4 from a VSVΔ51 platform did not induce drastic changes in the immune tumour microenvironment when compared to virus control in the presence or absence of GSK126 treatment. Full NanoString data set of B16-F10 tumours (n= 2 per group) treated with PBS (Vehicle control), VSVΔ51-amiR-NTC control and VSVΔ51-amiR-4 treatment (1E8 pfu/mouse) alone or in combination with Captisol (vehicle control) or GSK126 (50 mg/kg) is included.
